# Rethinking global health research: towards integrative expertise

**DOI:** 10.1186/1744-8603-5-6

**Published:** 2009-07-30

**Authors:** Malcolm MacLachlan

**Affiliations:** 1 Centre for Global Health and School of Psychology, Trinity College, Dublin, Ireland

## Abstract

The Bamako Call for Action on Research for Health stresses the importance of inter-disciplinary, inter-ministerial and inter-sectoral working. This challenges much of our current research and postgraduate research training in health, which mostly seeks to produce narrowly focused content specialists. We now need to compliment this type of research and research training, by offering alternative pathways that seek to create expertise, not only in specific narrow content areas, but also in the process and context of research, as well as in the interaction of these different facets of knowledge. Such an approach, developing 'integrative expertise', could greatly facilitate better research utilisation, helping policy makers and practitioners work through more evidence-based practice and across traditional research boundaries.

## Research for health & the 'Inter-land'

The Bamako Call for Action on Research for Health [1] arose from a meeting of Ministers of Health, Education, Science & Technology, Foreign Affairs and International Cooperation, from 59 countries; as well as researchers, policy makers, civil society representatives, journal editors and development agencies. From 17–19th November, 2008, in Bamako, Mali, amid presentations on the conference theme of "Strengthening Research for Health, Development and Equity" the call was crafted, having benefited from recent regional 'feeder' meetings in Algiers, Bangkok, Copenhagen, Rio de Janeiro and Tehran. This Ministerial conference, which occurs once every four years, is agenda setting; on this occasion the agenda being to change the way in which health research is undertaken.

IJsselmuiden and Matlin [2] note that the scope of health research is broad, including, for example, biomedical and public health research, research on health policy and systems, environmental health, science and technology, operational research, as well as social science and behavioural research. However, they argue that the range of research needed to protect and promote health and reduce disease is in fact much broader than this: "the fields of interest span the relationships between health and, among many others, social, economic, political, legal, agricultural and environmental factors" (p.4). For example, major health gains have been made possible through civil engineering improvements in water quality, sanitation and housing conditions, in addition to medicines and healthcare. As stated in the Bamako call itself:

"The nature of research and innovation for health improvement, especially in the context of the United Nations Millennium Development Goals, is not sufficiently inter-disciplinary and inter-sectoral; there is a need to mobilize all relevant sectors (public, private, civil society) to work together in effective and equitable partnerships to find needed solutions." (Recognition Statement 5)[1].

This mammoth revision of how research is conducted in our field could beckon a new age of enlightenment, but one for which we are currently poorly prepared. I argue that there is a need to rethink our approach to research in global and public health and to complement narrow research specialisations with a new cadre of researchers who have expertise concerning the context and process of research, as well as its content, and the interplay of these knowledge domains.

## Polymaths

The Renaissance was an age of enlightenment because it questioned previously narrow understandings of the world. It allowed the activities of questioning and discovery to be respectable, exciting and relevant to the curiosities and the exigencies of people's contemporary lives. Luminaries of that age were often distinguished not only by a genius in particular fields of interest, but also by their ability to traverse different fields of specialisation, and to sometimes recognise their interconnections. The 'polymaths' of Victorian times were also fired by broad curiosities, the delight of discovery and a hunger to better understand how the world worked, and indeed, how they could better work the world.

Aristotle was perhaps one of the original polymaths, his work embracing philosophy, logic, biology, astronomy, rhetoric and literary criticism, among others. Benjamin Franklin, as well as being a famous politician, was also a journalist, revolutionary, scientist and inventor; he invented bifocal lenses. A more contemporary polymath might be Umberto Eco, who while writing best selling novels, is an expert on literature, medieval philosophy, and pop culture and also a professor of semiotics.

The holding of such broad interests is today still viewed with some awe by the lay person, but rather disparaged by most in the research community. Those with too many research interests are seen as suffering from a sort of fuzzy parallelism; a restless unfocused mind, wandering without much conviction, across what must be a confusing intellectual landscape – to them anyway. The modern penchant in scientific research is to narrow down interests and expertise, resolutely. Narrow tracts of laser beam breadth enhance the probability that enthusiastic, active and able researchers can claim the coveted 'leading international authority' status that is so important in producing 'high impact' research, loading onto university rankings and research incomes.

## 'Naive Experts'

Commercial, legal and indeed egotistic exigencies, have fuelled the cult of the narrow researcher, and helped to make it a reassuring and satisfying role for many. It is important to acknowledge that this approach to research has also made many valuable contributions, with many benefits for society. However, it is also ironic that the reification of personalised expertise has been accompanied by an apparent depersonalisation of the authority associated with it. As argued in Nagel's *The View from Nowhere *[3], that view (uncluttered by personal opinion, context or bias) is sought in the drive for objectivity. That is, a view 'un-socially constructed' or cluttered by mere human or earthly issues, rather echoing classical views of 'pure knowledge'.

Again, let me stress, our cadre of narrowly focused 'objective' researchers have an impressive pantheon of achievements. These 'naïve experts' flourish amidst a view that the world, and the knowledge we now need for it, is too broad, too advanced, too complex, for anyone to really understand, very well, more than just one thing. Doing one thing well, has been magnified even further by the extra brownie points awarded for doing it in just one way – using a particular research methodology, rather than being 'muddled' by mixed methods, the results of which may be alarmingly messy to integrate. It is important to stress that the term 'naive experts', as I use it here, is not meant to be in any way pejorative, but rather descriptive of experts who may lack knowledge – of the uses or limitations – of their own knowledge.

## Research utilisation

The downside to a system that encourages the productions of intentionally naïve experts is their collateral ignorance of other ideas, approaches and possibilities, and most crucially of the utility of their research. Relatively few researchers are engaged with the application, or utilisation, of the knowledge they produce. Indeed, possibly the most intellectually challenging task – of putting together research findings from diverse perspectives and then deciding what should be done as a result of them – is left to policy makers and practitioners, who often are poorly prepared to interpret the strengths and weaknesses of different (and sometimes contradictory) research. I believe that researchers have an obligation – even a moral obligation – to do more. In some cases, this may already be happening through a broadening of research initiatives, viz multi-disciplinary, inter-disciplinary and trans-disciplinary research. But I believe that we need more broadly minded and broadly skilled researchers to usefully bring such diversity together.

What sort of neo-polymaths might we now need? What could we do to enhance the likelihood of research utilisation? One well known approach to policy analysis stresses the importance of distinguishing between content, context and process [4]. Such an approach might also usefully be applied to integrating research about 'what' (content), with research about 'where' (context) and 'how' (process). To take a concrete example, consider the challenges presented by HIV/AIDS. Here *Content Knowledge *could involve an appreciation of critical issues ranging across immunology, stigma and adherence to medication, and the different approaches to researching these. *Context Knowledge *could require understanding how HIV/AIDS is patterned across society, its social epidemiology, especially with regard to gender, disability, age, place, and socioeconomic status. *Process Knowledge *could involve awareness of the systems of application that need to be involved in putting knowledge into action. For instance, providing anti-retroviral medication in Dublin requires working through different systems of delivery than doing so in Durban or New Delhi. Process knowledge is about how to effectively get content knowledge put into practice, in particular contexts.

To take another example, and perhaps one that fits in well with the 'health-interland' described in the Bamako Call, research on health needs to embrace, to a much greater extent, the challenges faced by people with disabilities. People with disabilities have health needs just as do people without disabilities. However, to attain the highest possible standards of health, those researching disability from a health perspective need to interact with and understand other domains – transport, education and employment, for example. For *Content Knowledge *on prosthetic device development to provide the gains that are possible, it needs to be understood through *Process Knowledge*. For example, how such services are set up – whether they are targeted through specific NGOs or if there is a well developed interface between community and rehabilitative healthcare services – may have implications for the provision, servicing and replacement of such devices, and therefore the mobility of their users. Equally, *Context Knowledge*, of say resources and attitudes towards people with disabilities, may play a significant role in the extent to which people with disabilities are included or excluded from mainstream society and other support services that must work together for their well-being [5].

Global health is a 'composite' field, comprised of biological, clinical and social health sciences, and complemented by other disciplines that are not explicitly 'health related', such as engineering or political science. Within this cauldron of global health, we do, of course, already have some people who work across conventional intellectual boundaries and practice niches. For instance, Paul Farmer's work on HIV/AIDS (*Content*), his socio-political analysis of power relations (*Context*), and his service delivery role in *Partners in Health *(*Process*) are invigoratingly and productively braded together. And there are certainly other people who combine complementary activities and perspectives, but these people tend to emerge individually, we don't have an explicit way of producing or encouraging such skills, or encouraging a more integrative orientation in general; and we don't have a structure for teaching it.

If the Bamako Call for Action on Research for Health is to get traction, then we will need to develop research and research training that more explicitly helps researchers to think through research content, context and process knowledge, and how these can interplay, and contribute to better health. We will also need to examine some of our own 'institutions' that facilitate a sort of 'broad-minded inertia': discipline specific research funding streams; high impact journals cultivating a narrow research focus; education programmes that blinker young researchers to the legitimacy of other perspectives.

## Conclusion

While we should not underestimate the challenges of working across traditional boundaries and demarcations [6] nor should we overestimate the value of research that ignores such challenges. We need a reconfiguring of knowledge, not simply a diminution or expansion of it. We need to know more about how things fit together and can be put to good use. We cannot simply hope for broad-minded health researchers to spontaneously spring forth. Research training at postgraduate level should stress the value of *integrative expertise *as well as recognising *depth expertise*. If we can begin to address this challenge then we can do much to promote research utilisation; by making our research more relevant to global health practitioners and policy makers, and to the aspirations in the Bamako Call.

## Competing interests

The author was funded by the EU as a 'European Union Expert Delegate' at the Global Ministerial Forum on Research for Health, Bamako, 2008.
